# Supplementary material for: Critical Care in SARS-CoV-2 Infected Pregnant Women: A Prospective Multicenter Study
Source: Biomedicines. 2022 Feb 17;10(2):475. doi: 10.3390/biomedicines10020475 (PMC8962376; doi:10.3390/biomedicines10020475)
Supplement: Supplementary file 1 [file biomedicines-10-00475-s001.zip › biomedicines-1529851 - Supplementary Table 1.pdf]

Supplementary Table S1. List of hospitals members of the Spanish Obstetric Emergency Group included in this study (n = 78).

HOSPITAL
AGSE Hospital Axarquia
Complejo Asistencial de León
Complejo Hospitalario A Coruña
Complejo Hospitalario de Jaén
Complejo Hospitalario San Millán y San Pedro
Complejo Hospitalario Universitario de Pontevedra
Complexo Hospitalario Universitario de Ourense
HM Hospital Nuevo Belén
Hospital Alto Guadalquivir
Hospital Álvaro Cunqueiro (CHUVI)
Hospital Arnau de Vilanova
Hospital Clínico de Santiago de Compostela
Hospital Clínico San Carlos
Hospital Clínico San Cecilio (Complejo Hospitalario Universitario de Granada)
Hospital Clínico Universitario de Valladolid
Hospital Clínico Universitario Virgen de la Arrixaca
Hospital Costa del Sol
Hospital d'Inca
Hospital de la Santa Creu i Sant Pau
Hospital de Poniente
Hospital de Santa Caterina
Hospital de Son Llàtzer
Hospital de Torrejón
Hospital de Vinalopó
Hospital del Mar
Hospital del Tajo
Hospital do Barbanza
Hospital Doce de Octubre
Hospital Donostia
Hospital General de L'Hospitalet
Hospital General La Mancha Centro
Hospital General Universitario de Ciudad Real
Hospital General Universitario Gregorio Marañón
Hospital General Universitario Santa Lucía
Hospital Infanta Margarita
Hospital Jerez de la Frontera
Hospital La Fe
Hospital La Línea
Hospital Parc Taulí
Hospital Puerta de Hierro
Hospital Quirón Pozuelo de Alarcón
Hospital Quirónsalud Málaga
Hospital Rafael Méndez
Hospital Regional Universitario de Málaga
Hospital Reina Sofía

Hospital San Pedro Alcántara
Hospital Sant Joan de Reus
Hospital Santa Ana
Hospital Universitari Dexeus - Grupo Quirónsalud
Hospital Universitari Germans Trias i Pujol
Hospital Universitario Araba-Txagorritxu
Hospital Universitario Central de Asturias
Hospital Universitario de Basurto
Hospital Universitario de Burgos
Hospital Universitario de Cabueñes
Hospital Universitario de Ceuta
Hospital Universitario de Ferrol
Hospital Universitario de Fuenlabrada
Hospital Universitario de Girona Doctor Josep Trueta
Hospital Universitario de Salamanca
Hospital Universitario de Tarragona Juan XXIII
Hospital Universitario de Torrevieja
Hospital Universitario Doctor Peset
Hospital Universitario Infanta Sofía
Hospital Universitario La Paz
Hospital Universitario Puerta del Mar
Hospital Universitario Río Hortega
Hospital Universitario Son Espases
Hospital Universitario Torrecárdenas
Hospital Universitario Virgen de las Nieves
Hospital Universitario Virgen de Valme
Hospital Universitario Virgen del Rocio
Hospital Universitario Virgen Macarena
Hospital Univesitario de Getafe
Hospital Univesitario Severo Ochoa
Hospital Viamed Santa Ángela de la Cruz
Hospital Virgen de la Concha
Hospital Virgen de la Luz
